# Time-Course Microarray Analysis Reveals Differences between Transcriptional Changes in Tomato Leaves Triggered by Mild and Severe Variants of Potato Spindle Tuber Viroid

**DOI:** 10.3390/v10050257

**Published:** 2018-05-15

**Authors:** Aneta Więsyk, Roksana Iwanicka-Nowicka, Anna Fogtman, Włodzimierz Zagórski-Ostoja, Anna Góra-Sochacka

**Affiliations:** 1 Institute of Biochemistry and Biophysics Polish Academy of Sciences, Pawinskiego 5A, 02-106 Warsaw, Poland; anetaw@ibb.waw.pl (A.W.); roxana@ibb.waw.pl (R.I.-N.); AnnaFogtman@ibb.waw.pl (A.F.); 2 Laboratory of Systems Biology, Faculty of Biology, University of Warsaw, 02-096 Warsaw, Poland

**Keywords:** viroid, potato spindle tuber viroid (PSTVd), microarray, transcriptome analysis, differentially expressed genes, nCounter analysis

## Abstract

Viroids are small non-capsidated non-coding RNA replicons that utilize host factors for efficient propagation and spread through the entire plant. They can incite specific disease symptoms in susceptible plants. To better understand viroid-plant interactions, we employed microarray analysis to observe the changes of gene expression in “Rutgers” tomato leaves in response to the mild (M) and severe (S23) variants of potato spindle tuber viroid (PSTVd). The changes were analyzed over a time course of viroid infection development: (i) the pre-symptomatic stage; (ii) early symptoms; (iii) full spectrum of symptoms and (iv) the so-called ‘recovery’ stage, when stem regrowth was observed in severely affected plants. Gene expression profiles differed depending on stage of infection and variant. In S23-infected plants, the expression of over 3000 genes was affected, while M-infected plants showed 3-fold fewer differentially expressed genes, only 20% of which were specific to the M variant. The differentially expressed genes included many genes related to stress; defense; hormone metabolism and signaling; photosynthesis and chloroplasts; cell wall; RNA regulation, processing and binding; protein metabolism and modification and others. The expression levels of several genes were confirmed by nCounter analysis.

## 1. Introduction

Viroids are circular, single-stranded, highly structured RNA molecules of approximately 250–400 nucleotides in length that do not encode any proteins or peptides [1,2,3]. They are classified into two families: the *Avsunviroidae*, in which members replicate in the chloroplast, and the *Pospiviroidae*, in which members replicate in the nucleus [4]. Potato spindle tuber viroid (PSTVd) is the type member of the family *Pospiviroidae*. Its RNA is folded into a rod-like secondary structure divided into five structural and functional domains, the central (C), pathogenic (P), variable (V) and terminal left (TL) and right (TR) [5,6,7]. Like other members of the *Pospiviroidae*, PSTVd is replicated through an asymmetric rolling circle mechanism by the DNA-dependent RNA polymerase II [8,9]. The infecting circular (+)-strand RNA is transcribed into oligomeric (−)-linear RNAs, which in turn serve as templates to generate oligomeric (+)-linear RNAs that are finally cleaved by a host RNase [10] and ligated into unit-length circular RNAs by the nuclear DNA ligase 1 redirected to ligate RNA substrates [11]. Viroid RNA moves from cell to cell through plasmodesmata [12] while systemically spreading through the phloem [12,13,14].

The severity of symptoms depends on the viroid strain (its RNA sequence), host species and cultivar, and environmental conditions, and can affect whole plants or specific organs, such as leaves, stems, flowers, fruits, seeds, roots or storage organs [15]. In a susceptible host, such as tomato (*Solanum lycopersicum* cv. “Rutgers”), PSTVd can cause a wide spectrum of symptoms, from no symptoms through mild and intermediate to severe and even lethal. The typical severe symptoms on “Rutgers” tomato are stunting, shortening of stems, severe epinasty and rugosity of leaves, and necrosis of the veins and stems. Mild symptoms primarily appear as subtle stunting and epinasty. In addition to these macroscopic changes, disruption of the plasma membrane and abnormalities of the chloroplast and cell wall have been observed in PSTVd-infected plants [16].

Study of the viroid-host interaction has indicated that the mechanism of viroid pathogenesis can be mediated by the viroid genome itself or by viroid genome-derived ss- or dsRNAs that interact with host factors such as proteins or nucleic acids [17,18,19,20,21,22,23].

For example, PSTVd interaction in vitro with ribosomal protein L5 [24], protein kinases [25,26], Nt-4/1 [27,28], or DdRp II [8], and in vitro and in vivo with histones [29], VirP1 (viroid-binding protein 1) [30,31,32,33], transcription factor TFIIIA [24,34] or DNA ligase1 [11] have been demonstrated. Other interactions in vitro between eEIF1A (elongation factor 1-alpha) and PSTVd, CEVd (citrus exocortis viroid) and PLMVd (peach latent mosaic viroid) [35,36], phloem lectins with ASBVd (avocado sunblotch viroid) and HSVd (hop stunt viroid) (also in vivo) [37,38,39], and tRNA ligase [40], PARBP33 and PARBP35 (chloroplast RNA-binding protein) (also in vivo) [41] with ASBVd have been shown.

Transcriptional profiling analyses have revealed that viroid infections have a global effect on plant gene expression. These studies include PSTVd infection in tomato [23,42,43,44] and potato [45], CEVd [46] and CVd-III (citrus viroid III) [47] infection in Etrog citron, PLMVd infection in peach [48], HSVd in hop [49] and cucumber [50], and HLVd (hop latent viroid) and CBCVd (citrus bark cracking viroid) in hop [51]. The genes altered during PSTVd infection in tomato are mainly connected with defense, stress response, cell wall structure, chloroplast function, protein metabolism and hormone signaling pathways.

Viroids, as a unique class of non-coding RNA pathogens, provide a simple experimental system to study the direct impact of pathogenic RNA on a plant host. Despite previous studies, many unanswered questions remain regarding the mechanism of viroid pathogenesis. The identification of host genes in which expression is altered upon viroid infection could be helpful for understanding the processes determining plant growth, development and defense mechanisms against viroids. In the present study, we used microarray technology to perform gene expression analysis over a time course of mild and severe PSTVd infection development in “Rutgers” tomato, which is a well known and established experimental system for viroid pathogenesis research. Transcriptomes of plants infected with PSTVd-M (mild strain) and PSTVd-S23 (severe strain) were compared at four time points starting at the pre-symptomatic stage, 8 days post inoculation (dpi), through early and full symptom appearance and the so-called “recovery” stage at 49 dpi. The microarray data were validated by an nCounter analysis, which was also used to estimate relative PSTVd (+) RNA level.

## 2. Materials and Methods

### 2.1. Plant Material and PSTVd Infection

Tomato (*Solanum lycopersicum*) cv. “Rutgers” was used for the experiments. Seedlings with true leaves were mechanically inoculated using carborundum [52] with recombinant pUC9 plasmids (2 μg/plant) carrying an infectious monomeric cDNA copy of the severe strain, PSTVd-S23 (GenBank: X76846), or the mild strain, PSTVd-M (GenBank: X76844). Control plants were inoculated with the empty pUC9 plasmid. The inoculated plants were maintained in a greenhouse at temperatures 28–30 °C for 16 h and at 25 °C for 8 h in the dark, with a mean humidity of 55%. Symptom development was monitored daily from the initial day of inoculation up to 49 dpi.

### 2.2. PSTVd Detection

Viroid infection in the inoculated plants was verified by dot-blot hybridization with a PSTVd-specific digoxigenin-labeled RNA probe (DIG RNA labeling mix, Roche Diagnostics, Mannheim, Germany). Selected plant samples were additionally subjected to RT-qPCR analysis. One microgram of purified total RNA was reverse transcribed with a specific primer complementary to the CCR region (nucleotide positions in PSTVd-S23: 78–94) using the Omniscript RT Kit (Qiagen GmbH, Hilden, Germany) according to the manufacturer’s instructions. Real-time PCR was carried out in a PikoReal Real-Time PCR System (Thermo Scientific, Vilnius, Lithuania) using Luminaris Color HiGreen qPCR Master Mix (Thermo Scientific, Vilnius, Lithuania) and two specific primers, (i) CTTTCTTCGGGTGTCCTTCC and (ii) CTCGGGAGCTTCAGTTGTTTC, which were used previously by [53]. The cycling parameters were as follows: 1 cycle at 95 °C for 10 min, and 40 cycles each consisting of 15 s at 95 °C, 30 s at 59 °C and 30 s at 72 °C. Linearized plasmid encoding PSTVd-S23 was used to generate a standard curve. Data analysis was carried out by PikoReal™ Software 2.2.

### 2.3. RNA Isolation

Tomato leaf samples were collected from the same plants at four time points (8, 17, 24 and 49 dpi), each time from the 3rd–4th leaf from the apical bud. Samples were homogenized using a TissueLyser LT bead mill (Qiagen), and total RNA was extracted using the RNeasy Plant Mini Kit (Qiagen) following the manufacturer’s protocol. Contaminating DNA was removed by digestion with TURBO™ DNase (Ambion, Austin, TX, USA).

### 2.4. Microarray Analysis

Affymetrix technology (https://www.thermofisher.com) and Partek Genomics Suite Software (http://www.partek.com) are well world-wide established methods and are routinely used in Corelab (www.corelab.pl) [54,55,56], where microarray analysis was performed.

Three biological replicates of each of the three treatments (S23, M or pUC9 vector) at each time point derived from individual plants were processed independently, with one exception: due to technical problems, only two biological replicates of M-infected plants at 8 dpi were analyzed. Aliquots of 100 ng total RNA that passed the quality control screening were used to synthesize double-stranded cDNA. This reaction, the subsequent biotin-labeled cRNA synthesis, and fragmentation were performed using the 3′ IVT PLUS reagent kit according to the manufacturer’s instructions (Affymetrix, Santa Clara, CA, USA). Labeled samples were hybridized to the GeneChip^®^ Tomato Genome 1.0 ST Array (Affymetrix, Santa Clara, CA, USA). The microarrays were scanned with the Affymetrix GeneAtlas Scanner, and the intensity signals for each probe set were recorded by the Affymetrix software as CEL files. The CEL files were imported into Partek Genomics Suite v 7 software with the use of RMA (robust multiarray averaging) [57]. During this step, background correction was applied based on the global distribution of the PM (perfect match) probe intensities, and the affinity for each of the probes (based on their sequences) was calculated. Then, the probe intensities were quantile normalized [58] and log_2_ transformed, and median polish summarization was applied to each of the probe sets. Then, the qualitative analysis was performed, e.g., principal component analysis [59], in order to identify outliers and artifacts on the microarray. After quality checking, a 3-way analysis of variance (ANOVA) model using method of moments [60] was applied to the data, which allowed the creation of lists of significantly differentially expressed genes (DEGs) between the biological variants (cutoffs: *p* value < 0.05, −2.0 ≥ fold change (FC) ≥ 2.0).

The complete datasets of the microarray experiment are available in the NCBI Gene Expression Omnibus (GEO) database repository with accession number GSE106912.

### 2.5. Microarray Data Analysis

Affymetrix probes were aligned to the released version of the tomato genome sequence ITAG 2.4 (http://solgenomics.net). Gene Ontology (GO) annotation was obtained using Blast2GO software [61]. The list of up- and down-regulated genes was searched for overrepresented GO terms, and *p* values were computed with Fischer’s exact test and corrected for multiple testing; a *p* value ≤ 0.005 was considered statistically significant. Pathway annotation was performed at Kyoto Encyclopedia of Genes and Genomes (KEGG) using the KEGG Automatic Annotation Server (KAAS, http://www.genome.jp/kegg/kaas/) [62]. Venn diagrams were generated using the Venny 2.1.0 online tool (http://bioinfogp.cnb.csic.es/tools/venny/index.html) [63].

### 2.6. NanoString nCounter Analysis

Selected RNA samples (150 ng) used in the microarray analyses were additionally subjected to NanoString nCounter^®^ multiplex gene expression analysis (NanoString Technologies Inc., Seattle, WC, USA, www.nanostring.com) at the Core Facility Molecular Biology in Medizinische Universität Graz [64,65]. An nCounter CodeSet was designed for 19 DEGs, 3 housekeeping genes and viroid RNA (Table S1). The obtained nCounter data were adjusted according to the manufacturer’s instructions using the manufacturer-provided spiked positive and negative controls. The geometric means of the three housekeeping genes: *alpha glucosidase II* (*gluII*, NM_001247101.2), *glyceraldehyde 3-phosphate dehydrogenase* (*GAPDH*, NM_001247874.2) and *actin* (NM_001321306.1) were used to normalize gene expression value for the tested genes and viroid titer. Spearman rank correlation [66] was performed using STATISTICA v. 12 software (StatSoft Inc., Tulsa, OK, USA) to compare the expression data from the nCounter and microarray analyses for the 19 genes.

### 2.7. Sequencing of the PSTVd-M and PSTVd-S23 Progeny

RT-PCR was performed with purified RNA using two specific primers corresponding to the CCR region [67], the Omniscript Reverse Transcriptase kit (Qiagen) and Easy-A High-Fidelity PCR Master Mix (Stratagene, San Diego, CA, USA) . The PCR products were directly sequenced, and the obtained sequences were analyzed using Lasergene software (DNASTAR Inc., Madison, WI, USA).

## 3. Results

### 3.1. Time-Course of Symptom Development

**Figure 1:**
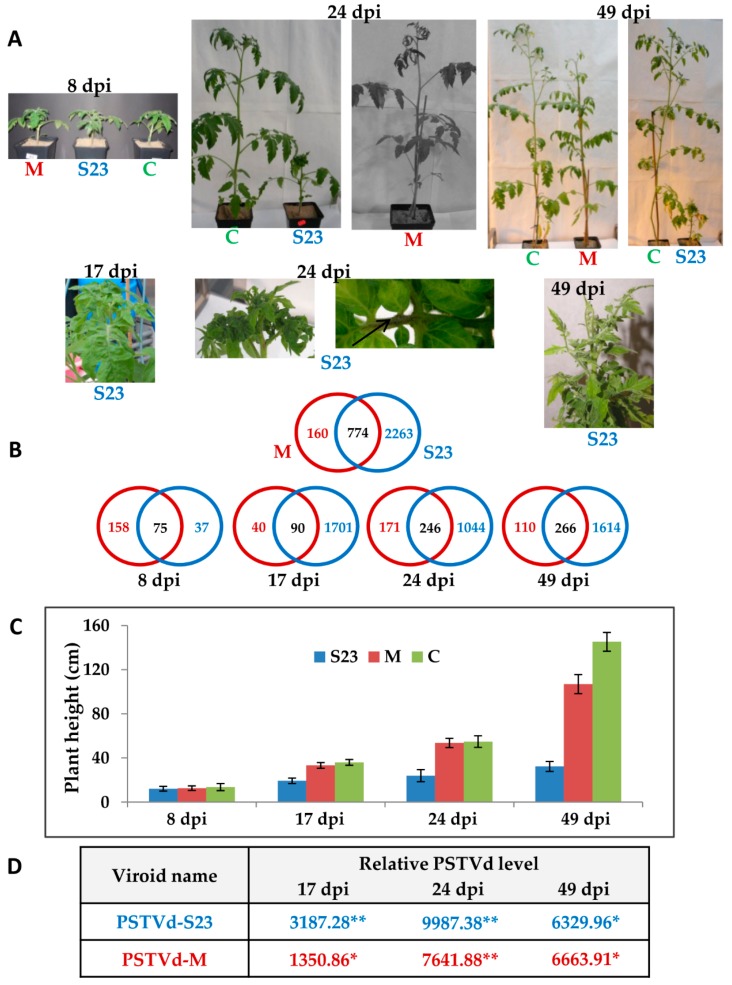
Time course of symptom development of PSTVd infection on “Rutgers” tomato in correlation with viroid RNA level and number of DEGs. (**A**) Comparison of symptoms induced by the mild and severe PSTVd variants. Typical necrosis caused by S23 at 24 dpi are indicated by arrow. (**B**) Venn diagrams of the DEGs: total DEGs in M and S23 infected plants, and at the indicated time points. (**C**) Comparison of plant heights at 4 time points. Each bar represents the arithmetic mean of the height of three plants with SD indicated. C, control plant (pUC9 vector-inoculated). (**D**) Relative viroid level measured by the NanoString nCounter method. Relative viroid level in arbitrary units in comparison to control plants was estimated using nSolver analysis software (v. 3.0, NanoString Technology, Seattle, WC, USA). A *t*-test was used to determine the significance of the differences; * and ** indicate *p* < 0.05 and *p* < 0.001, respectively.

For a comparative analysis of the changes in “Rutgers” tomato transcript profiles in response to mild and severe PSTVd infection, we selected two well characterized strains. The first, PSTVd-S23, induces typical severe symptoms such as strong stunting, leaf curling and epinasty, necrosis and chlorosis, while the second, PSTVd-M, induces only mild stunting and epinasty (Figure 1) At the molecular level, these variants differ at nine nucleotide positions located in the P and V domains [67].

Following PSTVd inoculation, leaf samples for RNA isolation were collected at 4 time points corresponding to the subsequent disease stages: (i) pre-symptomatic, 8 dpi; (ii) early stage of symptoms (observed in only S23-infected plants), 17 dpi; (iii) full spectrum of symptoms, 24 dpi; and (iv) “recovery” (regrowth of stem observed in S23-infected plants), 49 dpi (Figure 1). PSTVd infection was confirmed by dot-blot hybridization in samples from all disease stages but could not be confirmed at the pre-symptomatic stage (8 dpi), even by a more sensitive method such as qRT-PCR. At 17 dpi the level of S23 RNA was two fold higher than that of the M RNA. In both infections, statistically significant increase in PSTVd RNA level was observed until 24 dpi. Between 24 and 49 dpi viroid level was more or less stable and comparable in both strains (Figure 1). The observed decrease of S23 RNA level at 49 dpi was not statistically significant. Additionally, direct sequencing of RT-PCR products confirmed the presence of the PSTVd-S23 and PSTVd-M variant sequences in plants at 17 and 49 dpi.

### 3.2. Overview of Time-Course Analysis of the Tomato Transcriptome in Response to Mild and Severe PSTVd Variants

**Figure 2:**
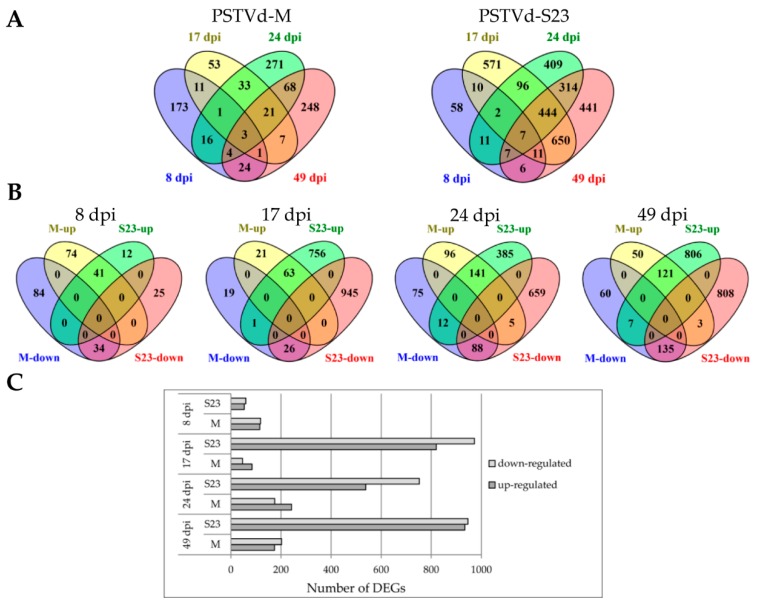
Relationship of DEGs at different time points. (**A**) Venn diagrams display the distribution of DEGs at each time point during mild or severe infection. (**B**) Venn diagrams display the distribution of up- and down-regulated genes in both infections at each time point. (**C**) Comparison of the number of up- and down-regulated genes at each time point.

Using microarray technology, we performed leaf transcriptome profiling assays on mock-inoculated, PSTVd-S23 and PSTVd-M-infected plants and compared them at four time points. Using the criteria of *p* value < 0.05 and FC ≤ −2.00 or ≥ 2.00, a total of 3197 genes of the nearly 10,000 genes represented on the Affymetrix Tomato GeneChip were significantly altered in PSTVd-infected plants at one or more of the four time points (Table S2, Figure 1). Of the 3197 identified DEGs, 3037 were observed in S23 infection, while less than one third that number were found in mild infection. Overall, 160 and 2263 DEGs were specific to mild and severe infections, respectively, while 774 DEGs were observed in both cases (Figure 1). Different ratios of up- and down-regulated genes were observed depending on disease stage and viroid variant (Figure 2). Interestingly, as shown in the Venn diagrams (Figure 2A), the highest number of common DEGs in the S23 infection was found between day 17 and day 49, i.e., at the stage when symptoms became visible and the stage when stem regrowth is observed. In the mild infection, days 24 and 49 shared the highest number of DEGs, and both these time points are correlated with the expression of symptoms, i.e., mild epinasty and stunting.

A total of 3197 DEGs were organized into functional categories according to the GO guidelines (Gene Ontology Consortium, 2000) using Blast2GO. In total, 841 and 2764 DEGs in M- and S23-infected plants, respectively, were annotated with at least one GO annotation (Figure S1). This analysis did not indicate an essential difference between mild and severe infection.

### 3.3. Functional Classifications of Genes Differentially Expressed at Three Time Points

Among the 3037 DEGs identified in severe infection, seven were common to all four time points. For example, these included serine threonine-kinase, leucine-rich repeat receptor-like serine/threonine/tyrosine-protein kinase SOBIR1 precursor and xyloglucan endotransglucosylase.

Exclusion of the first time point allowed for the identification of 444 genes that were common to the remaining time points. Several of these genes were differentially regulated depending on the time point, but 199 and 229 of these genes were constantly up-regulated or down-regulated, respectively, at all three time points, i.e., 17, 24 and 49 dpi.

Interestingly, of the 934 DEGs found in M-infected plants, only 3 genes (3-hydroxyisobutyryl-CoA hydrolase 1-like, cytochrome P450 CYP72A219-like and putative F-box protein At3g16210) and 21 genes overlapped four and three (17, 24 and 49 dpi) of the time points, respectively. Among these 21 DEGs, 15 and 5 were consistently up or down-regulated, respectively (Figure 2).

**Figure 3:**
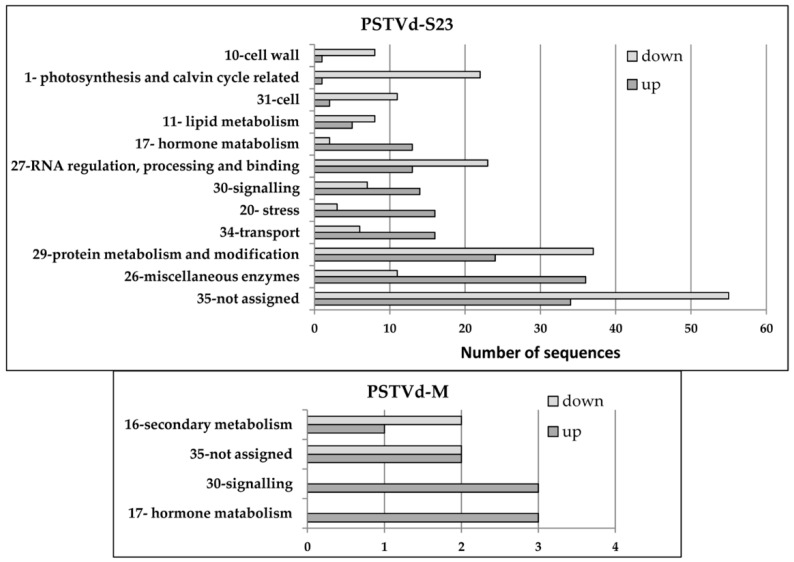
Functional analysis of DEGs commonly identified at three time points by MapMan. DEGs from the up- and down-regulated groups were assigned to 35 bins in the “Overview” visualization pathway of MapMan. The bin numbers and corresponding names are graphed on the Y-axis. Only the most abundant categories for at least one group of genes are presented. Miscellaneous enzymes, e.g., cytochrome P450, CCN5-related N-acetyltransferase, UDP glucosyl and glucuronyl transferases.

The distribution of the genes regulated at three time points (17, 24 and 49 dpi) into different functional groups by MapMan analysis is shown in Figure 3. Only genes related to hormone metabolism and signaling were commonly up-regulated in both infections during this time span.

### 3.4. GO Enrichment Analysis of the Identified DEGs at Each Time Point

**Figure 4:**
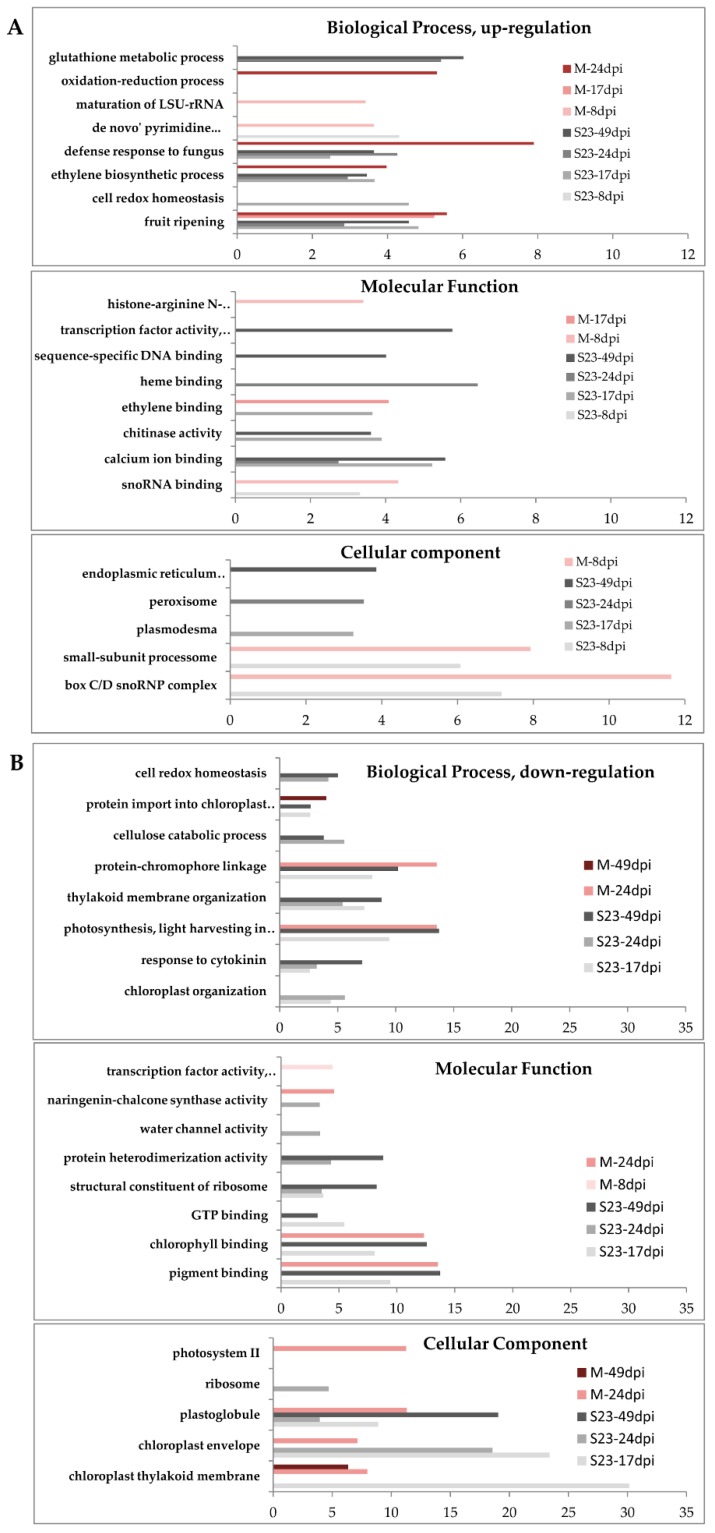
GO enrichment analysis. (**A**,**B**) up- and down-regulated genes, respectively. Only selected GO terms for the S23 and M variants at the indicated times after inoculation are presented. Categories are indicated on the Y-axis, log_10_(1/p) is on the X-axis; enrichment *p* value < 0.005 was used as a cutoff.

We performed GO enrichment analysis as implemented in the Blast2GO program with two sets of DEGs that were up- or down-regulated. The analysis was restricted to GO terms that were ranked lower in the GO hierarchy, referring to more specific processes, functions and localizations. All data can be found in Table S3, and selected data are also presented in Figure 4.

#### 3.4.1. Up-Regulated Genes

In the earliest S23 samples, only 5 GO terms were enriched among the up-regulated genes. These included, for example, snoRNA binding, “de novo” pyrimidine nucleobase biosynthetic process and box C/D snoRNP complex in the metabolic function (MF), biological process (BP) and cellular component (CC) categories, respectively. In the BP category, genes involved in toxin catabolism, fruit ripening, ethylene biosynthesis and defense response were overrepresented at 17, 24, and 49 dpi, while those involved, for example, in cell redox homeostasis and gibberellin biosynthesis were overrepresented at only one time point each, 17 and 24 dpi, respectively. Some enriched GO terms were also common to two time points, such as positive regulation of RNA polymerase II transcriptional preinitiation complex assembly at 17 and 49 dpi or glutathione metabolic process at 24 and 49 dpi. In the MF category, the enriched GO terms at three time points (17, 24 and 49 dpi) included, for example, calcium ion binding and glutathione transferase activity, while chitinase activity was overrepresented at both 17 and 49 dpi. At 24 dpi, 10 GO terms were indicated, and 6 of them, such as heme or iron ion binding and gibberellin 20-oxidase activity, were specific to this time point. In the CC category, 8 GO terms were overrepresented: for example, plasmodesma, peroxisome and endoplasmic reticulum membrane at 17, 24 and 49 dpi, respectively.

In the earliest M samples, several enriched terms were identified in the 3 main GO categories. Some of these terms were exclusively observed in mild infection, i.e., maturation of LSU-rRNA, mitochondrial intermembrane space protein transporter complex and histone-arginine *N*-methyltransferase activity in the BP, CC and MF categories, respectively. At 17 dpi and/or 24 dpi, the BP and MF categories showed changes in genes involved in fruit ripening, ethylene biosynthesis and binding, negative regulation of ethylene-activated signaling pathway or defense response. We did not observe any enrichment in the CC category at 17 or 24 dpi; moreover, at 49 dpi, no enrichment was observed in any category.

#### 3.4.2. Down-Regulated Genes

In the earliest S23 leaf samples, no significant enrichment was found in any major GO category for down-regulated genes. Between 17 and 49 dpi, many GO terms from the BP category were enriched, and most of these were common to two time points. The repressed genes were mainly connected with chloroplast organization (17 and 24 dpi), thylakoid membrane organization (17, 24 and 49 dpi), photosynthesis (light harvesting in photosystem I, 17 and 49 dpi), cellulose catabolism (24 and 49 dpi) or response to cytokinin (17, 24 and 49 dpi). In the categories of CC and MF, the overrepresented genes were mainly associated with chloroplasts, photosynthesis and ribosomes. Moreover, in the category MF at 17 and 49 dpi, the most enriched terms were pigment, chlorophyll and GTP binding, while at 24 dpi, cellulase activity and protein heterodimerization activity were the most enriched, and the same terms were also observed at 49 dpi.

In the mild samples, only 17 GO terms in the BP, MF and CC categories were enriched for at least one of the three time points 8, 24 and 49 dpi. Most of these terms overlapped those observed in the S23 samples.

Interestingly, at 24 dpi in both M- and S23-infected plants, the term GO naringenin-chalcone synthase activity was enriched among the down-regulated genes. Naringenin-chalcone synthase is a key enzyme for phenylpropanoid synthesis, and its down-regulation was also observed in hop plants infected with HSVd and CPFVd (cucumber pale fruit viroid) [68].

### 3.5. Differential Regulation of Genes Encoding Transcription Factors and Protein Kinases

Transcription factors (TFs) and protein kinases (PKs) were identified based on the data deposited at http://bioinfo.bti.cornell.edu/cgi-bin/itak/index.cgi [69]. PK classification according to [70] was used.

#### 3.5.1. Transcription Factors

**Figure 5:**
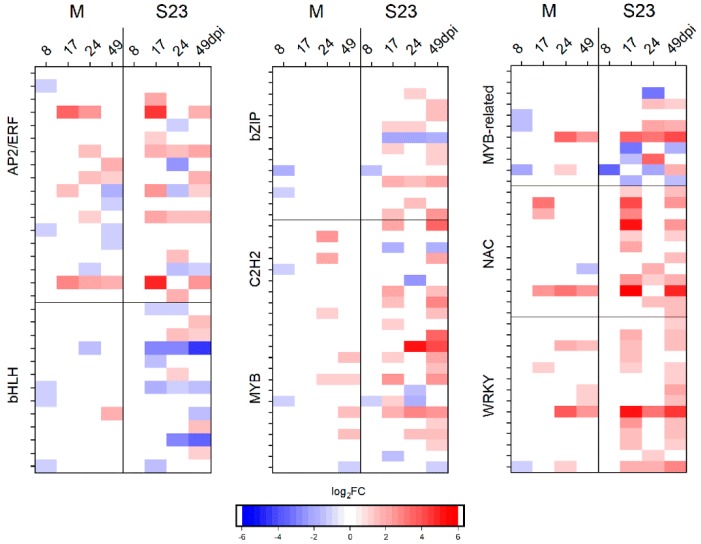
Heat map of log_2_ fold change of genes encoding transcription factors belonging to the indicated families.

Among the genes differentially regulated during S23 and M infection, 165 and 75 genes, respectively, were classified as TFs. In S23, these TFs belonged to 41 families, with the highest number annotated in the families AP2/ERF-ERF, bHLH, bZIP, C2H2, MYB, NAC and WRKY. In M, TFs from 29 families were recognized, with the highest number found in the families ERF, bHLH, Myb, Myb-related, WRKY, NAC and C2H2 (Table S4, Figure 5).

Factors from the ethylene-responsive factor (ERF) family play important roles in many cellular processes such as hormonal signal transduction, response to biotic and abiotic stresses and regulation of metabolism [71]. Upon viroid infection, the expression of 17 genes encoding ERFs, Pti4, Pti6 and dehydration-responsive element-binding proteins was altered. Most of them were up-regulated.

In the bHLH family, out of 13 altered genes, 4 were associated with both mild and severe infection. One and 8 genes were exclusively altered in mild and severe infection, respectively. Most of the altered bHLH genes were down-regulated.

TFs from the bZIP family (basic leucine zipper) are key regulators of growth and development and are functionally involved in oxidative stress [72,73]. Most of the identified genes were present in only the S23-infected plants.

C2H2 zinc finger (C2H2-ZF) proteins constitute a large gene family in plants and participate in various aspects of normal plant growth and development, as well as in biotic and abiotic stress responses. We identified 11 genes altered by viroid infection, most of them up-regulated. One protein indeterminate-domain 9 gene and two other genes, zinc finger protein 3-like and transcription factor IIIA, were down-regulated in M and S23, respectively. Based on the in vitro binding capacity of TFIIIA from Arabidopsis thaliana, it was proposed that TFIIIA could act as a bridge between the viroid RNA template and polymerase II [24]. Later, in Nicotiana benthamiana, suppression of TFIIIA (variant 7Z lacking first two ZFs) was shown to reduce PSTVd replication while overexpression enhanced PSTVd replication in planta [34]. In our experiment conducted in a different plant host, TFIIIA was down-regulated only in S23-infected plants at 17 and 49 dpi but not at 24 dpi when the viroid RNA level was higher. Searching of DEG lists from published data indicated no regulation of the TFIIIA gene in tomato infected with an intermediate PSTVd strain at 28 dpi [43] or 21 dpi [23]. This result is consistent with our data for the mild variant (and severe at 24 dpi) and indicates differences among transcriptional profile changes after infection with strains of different virulences as well as differences among the stages of viroid infection.

Myb family TFs control many physiological processes, including biotic and abiotic stress responses. We observed altered expression of 12 Myb genes; half of them were specific to S23 infection, and the rest common to both strains.

WRKY TFs play an important role in the regulation of gene transcriptional reprogramming in plant stress responses [74]. Overall, the altered genes, 8 exclusive to S23 infection and 6 common for both strains, were up-regulated.

The NAC family plays roles in plant growth and development, including responses to abiotic stress [75,76]. Higher up-regulation was observed in S23-infected plants than in M-infected plants.

#### 3.5.2. Protein Kinases

Expression alterations of over 70 PKs were observed during S23 infection, compared to 29 during M infection. These PKs were assigned to over 20 families (Table S4).

### 3.6. Genes Related to Jasmonic Acid, Ethylene and Salicylic Acid Biosynthesis and Signaling

**Figure 6:**
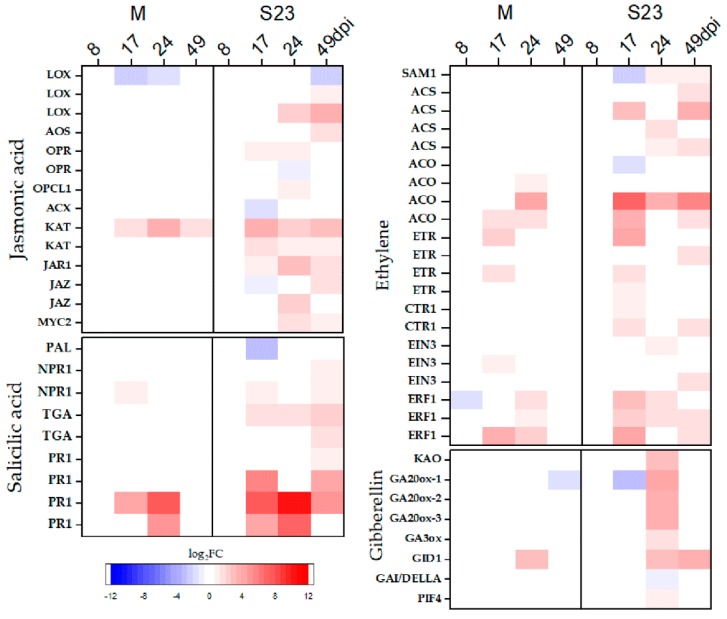
Heat map of log_2_ fold change of DEGs involved in plant hormone biosynthesis and signaling. Only selected hormones are presented.

As shown in Table S5 and Figure 6, the expression of several genes involved in JA biosynthesis and signaling was altered, mostly by the S23 variant. One *LOX* (lipoxygenase) gene was down-regulated in M-infected plants at 17 and 49 dpi, while one *KAT* gene (3-ketoacyl-CoA thiolase) was up-regulated between 17 and 49 dpi. In addition to the down-regulation of three genes, *LOX* (the same as in M infection), *OPR* (12-oxophytodienoate reductase 3), and *ACS* (acyl-CoA oxidase), other genes related to biosynthesis were up-regulated by PSTVd-S23. Similarly, in the perception and signaling pathway, genes encoding Jasmonate-Zim Domain (JAZ) protein and MYC2 TFs were up-regulated during S23 infection.

Ethylene (ET) is synthesized from methionine through the sequential action of three enzymes: *S*-adenosyl-l-methionine synthetase (SAM1), 1-aminocyclopropane-1-carboxylate (ACC) synthase (ACS) and ACC oxidase (ACO). The *ACO* and *ACS* genes were up-regulated at various time points in both PSTVd infections, while the expression of the *SAM1* gene was reduced (FC = −6.3) at 17 dpi but later increased several fold (FC = 2.4) upon S23 infection. Genes coding for proteins engaged in ET signaling, such as ER-localized receptors (ETR), constitutive triple response1 (CTR1), the transcription factor EIN3 and ERF, were up-regulated in both M- and S23-infected plants, but no differential regulation was observed at 49 dpi in M infection (Figure 6, Table S5).

We observed only down-regulation in S23 at 17 dpi of the gene encoding phenylalanine ammonia-lyase (PAL), the key enzyme in salicylic acid (SA) biosynthesis. However, genes coding for proteins belonging to the SA signaling pathway, such as the regulatory protein NPR1 and the transcription factor TGA, were up-regulated beginning at 17 dpi (Figure 6, Table S5).

### 3.7. Genes Related to Gibberellin, Abscisic Acid, Brassinosteroid, Cytokinin and Auxin Biosynthesis and Signaling

Gibberellins (GA), abscisic acid (ABA), brassinosteroids (BR) and cytokinin (CK) are isoprenoid derivatives [77]. In our experiments, we observed down-regulation of most genes encoding the enzymes associated with the biosynthesis of isoprenoid at 17 dpi (7 genes) and 49 dpi (5 genes) during S23 infection and at 24 dpi (3 genes) during M infection.

The expression of genes involved in the GA biosynthetic process, ent-kaurenoic acid oxidase (KAO), GA20-ox (3 genes) and GA3-ox, was increased at 24 dpi in plants infected with S23. One of the GA20-ox genes (Solyc03g006880.2.1) was down-regulated at 17 dpi and 49 dpi in S23 and M infection, respectively. The expression of genes encoding the nuclear receptor GID1, DELLA protein, and PIF 4 transcription factor, which are involved in GA signaling [78,79,80], were mostly altered in the severely infected plants. Genes coding for GID1 and PIF 4 were induced at 24–49 dpi and 24 dpi, respectively, whereas a decrease in the transcript level of the genes coding for DELLA proteins was observed at 24 dpi. In M-infected plants, only the induction of GID1 at 24 dpi was observed (Figure 6).

Three genes that belong to the LONELY GUY (LOG) family, encoding enzymes that convert inactive cytokinin to an active form [81], were altered in only the plants infected with S23. One of three genes coding for zeatin O-glucosyltransferase (CISZOG), which inactivates active CK, was highly up-regulated at 17 and 49 dpi in PSTV-S23 infected plants. The second was down-regulated at 17 dpi in M-infected plants, and the third one was down- and up-regulated (at 24 and 49 dpi) during S23 infection. In CK signal transduction, up-regulated transcription of *AHP* genes (histidine-containing phosphotransfer protein) was observed in S23-infected plants. AHPs transmit phosphorylation signals to response regulators (ARRs) in the nucleus, which in turn coordinate target gene transcription and feedback modulation of the signaling cascade [82]. Type-A ARRs acting as repressors of CK signaling provide an efficient instrument to modulate, dampen or shut down CK output in response to a change in the signal input or through crosstalk with other pathways [83]. In general, in M and S23 infection, the expression of genes coding for type-A ARRs was decreased at 17 dpi and increased at 24 and 49 dpi (Table S5).

Most genes encoding enzymes involved in ABA biosynthesis were down-regulated in S23-infected plants, especially at 17 and 49 dpi, with the exception of the phytoene synthase 1 gene, which was up-regulated in S23 at 17 dpi and 49 dpi and in M infection at 24 dpi. Most genes involved in ABA signaling were altered at 17 dpi in S23; for example, three *PP2C* genes and the *PYR/PYL/RCAR* gene (Solyc03g007310.2.1) were down-regulated. At 8 dpi, a decreased level of *ABF* (abscisic acid responsive element-binding factor) gene expression in both M- and S23-infected plants was observed. Moreover, in plants infected with S23, a constant increase in the level of *SnRK2* transcript was observed, starting from 17 dpi. The plastid-localized ABA receptor is the H subunit of Mg chelatase (CHLH), which binds a group of WRKY TFs (WRKY40, 18, and 60) in the presence of ABA, preventing them from moving to the nucleus, where they would otherwise repress the expression of several ABA-response loci. We observed an increased level of transcripts coding for WRKY40 and a decreased level of transcripts coding for CHLH, and this alteration in expression level appeared earlier in plants infected with S23 (17 dpi) than in those infected with M (24 dpi).

In the S23-infected plants, the expression levels of genes involved in sterol synthesis were reduced at 17 and 49 dpi but up-regulated at 24 dpi. In the direct brassinosteroid biosynthesis pathway, genes encoding cytochromes P450 724B1 and P450 90A1 were down-regulated. However, 3 other genes were up-regulated, and 2 of these were also up-regulated in the M variant. One of these genes (*CYP92A6*) was very highly up-regulated, i.e., FC = 133 and FC = 43 in S23 (17 dpi) and M (24 dpi) infection, respectively. Notably, genes involved in signal transduction were down-regulated in S23 infection at 17–49 dpi and in M infection at 24 dpi (Table S5).

Two major pathways, the tryptophan (Trp)-independent and Trp-dependent, have been proposed for the biosynthesis of indole-3-pyruvic acid (IAA) [84], and these pathways were altered in our study, predominantly in severe infection starting at 17 dpi. Additionally, in the signaling pathway, more genes with altered expression were observed in S23 infection, e.g., *AUX/IAA* (auxin-responsive protein IAA), *GH3* (auxin-responsive GH3 gene family), and *SAUR* (small auxin up-regulated RNA). AUX/IAAs are negative repressors that bind to the ARFs (auxin-responsive factors) of the target genes, and their degradation is promoted by auxin. All but *AUX/IAA26* were down-regulated in both S23 and M infection. Down-regulation was also observed in the *ARF9* and *ARF3* genes. The expression of the gene encoding LAX1, which is involved in auxin transport, was reduced at 17 and 24 dpi in only the S23-infected plants (Table S5).

### 3.8. Genes Related to Photosynthesis and Chloroplasts

In addition to photosynthesis, chloroplasts take part in the synthesis of numerous compounds including secondary metabolites, fatty acids, amino acids and important mediators of plant immune responses such as hormones (SA, JA, and ABA) and secondary messengers including calcium and reactive oxygen species (ROS). They also produce retrograde signals to alter nuclear gene expression in order to regulate chloroplast biogenesis, maintain homeostasis, or optimize chloroplast performance in response to developmental cues and stresses including pathogen attack [85].

Previous studies using microarray, macroarray and RNA-seq approaches revealed reduced expression of chloroplast- and photosynthesis-related genes in plants during viroid infection [42,43,46,49,50]. Moreover, measurements of photosynthetic rate demonstrated photosynthesis inhibition in cucumber plants infected with HSVd [50]. In our study, among the 37 DEGs involved in the light reaction, 36 genes were down-regulated (11, 13 and 33 genes at 17, 24, and 49 dpi, respectively) in S23-infected plants, but only 5 were down-regulated in M-infected plants at one time point (49 dpi). Moreover, down-regulation of genes encoding chlorophyll a/b binding proteins was observed in S23-infected plants predominantly at 17 and 49 dpi, while in M-infected plants, this down-regulation occurred at 24 dpi. Twelve genes functioning in the Calvin-Benson cycle were down-regulated and 2 were up-regulated starting at the 17th day of S23 infection, and 5 such genes were down-regulated at 49 dpi in M-infected plants. We also observed decreased expression of genes implicated in chlorophyll metabolism, mainly during severe infection. Interestingly, the expression of the *SGR* (magnesium dechelatase) and *RCCR* (red chlorophyll catabolic reductase) genes, which encode enzymes associated with chlorophyll degradation, were decreased, in contrast to an increased level of *PAO* (pheophorbide a oxygenase), at 24 dpi. All data are presented in Table S6.

### 3.9. Cell Wall-Related Genes

We observed altered transcript levels of many genes related to cell wall biosynthesis and organization (Table S7). These genes included those involved in the synthesis and remodeling of cell walls, such as cellulose synthase, endo-1,4-β-glucanases, pectinesterase, and pectate lyase. Moreover, the expression of genes encoding the structural proteins of the cell wall, e.g., extensins, arabinogalactan-proteins, proline-rich proteins (PRPs), and glycine-rich proteins (GRP), as well as expansins, was altered. Genes encoding arabinogalactan and PRP were strongly down-regulated between 17 and 49 dpi in S23 infection, whereas decreased expression of only a few genes was observed in M only at 24 dpi.

Lignin biosynthesis can be induced by various biotic and abiotic stress conditions and by perturbation in the cell wall structure [86,87]. We observed alterations in the expression of some genes connected with lignin biosynthesis, and most of these were found in plants with severe disease symptoms. A reduced expression level of the gene coding for phenylalanine ammonia-lyase (PAL), the enzyme responsible for the first step in the phenylpropanoid pathway, was observed at 17 dpi. Further along in the same pathway, the levels of the genes encoding 4-coumarate-CoA ligase (4CL) and caffeoyl-CoA O-methyltransferase (CCoAoMT) and 2 of the 3 genes for cinnamyl-alcohol dehydrogenase (CAD) were up-regulated, mostly at 17 and 49 dpi. Interestingly, the gene (Solyc11g011340.1.1) coding for CAD showed reduced expression in M-infected plants at 8 dpi (FC = −54), whereas an increase was found in S23 plants at 17 and 24 dpi (FC = 21 and 27, respectively).

## 4. Validation of Microarray Data

**Figure 7:**
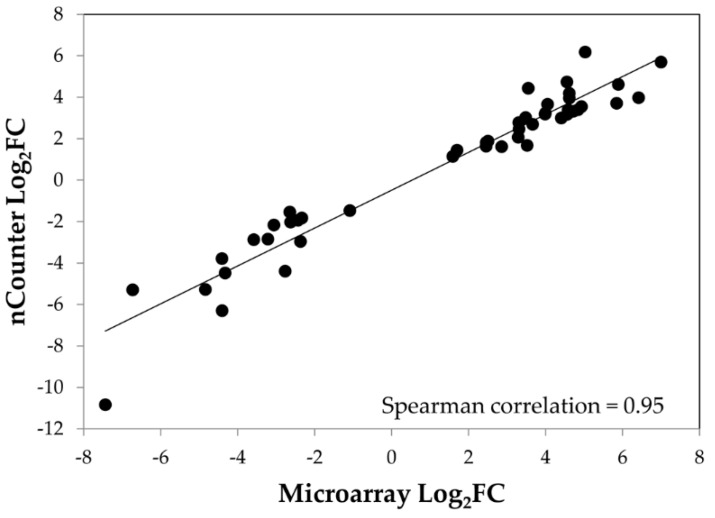
Correlation of microarray and nCounter NanoString data for selected DEGs. The Spearman rank correlation of the log_2_ differences between the microarray and NanoString nCounter*^®^* measurements for the 19 selected DEGs is presented. The mean of the log_2_FC value between the PSTVd-infected and mock-inoculated samples was calculated from three or two biological replicates.

To validate the results of the microarray analysis, we used nCounter Technology, which is based on direct multiplex measurement of gene expression and offers high levels of precision and sensitivity. Specific probes were designed for 19 selected genes (Table S1) previously identified in the microarray analysis as up- or down-regulated to various degrees depending on the time point and PSTVd variant. The genes of interest included genes involved in various pathways such as hormone metabolism and signaling, defense response or TFs. For the 19 selected genes, we observed a strong correlation between these two methods (Spearman correlation = 0.95; Figure 7, Table S1). Some of these genes are discussed further below.

## 5. Discussion

In this study, we used microarrays to analyze the transcriptomic changes in tomato leaves following infection with two PSTVd variants with significantly different virulence. Collecting samples at four time points from the same plants and using leaves from similar locations (3–4 leaves from the apical bud) allowed us to follow the development of systemic viroid infection. Different dynamics were observed during the development of mild and severe infections, and their correlations with the dynamics of plant defense response also differed. In S23 infection, a sharp increase in DEG number was observed at 17 dpi, followed by a slight decrease at 24 dpi and another increase at 49 dpi. In M-infected plants, the DEG number fluctuations were milder and in the opposite directions to those in S23. Unlike the other stages, the pre-symptomatic stage (8 dpi) showed a higher number of DEGs in M-infected plants (Figure 1B and Figure 2A,C, Table S2), and the majority of these DEGs were exclusive to the M variant. Among them defensin-like protein P322 was highly activated only in M-infected plants. This preliminary evidence is very interesting, but a more detailed analysis of viroid levels between day 8 and day 17 will be required.

Overall, the number of DEGs recognized in the mild infection was significantly lower than that in the severe infection (Figure 1B). Similarly, a macroarray analysis performed by Itaya and coworkers [42] revealed that the number of DEGs in severe PSTVd infection (55 genes) was two times higher (22 genes) than that in mild infection. Tsushima et al. [88] showed faster accumulation of a severe PSTVd isolate and higher relative levels of viroid-specific small RNAs and microRNAs than those of a mild isolate. Correlations between symptom severity and level of viroid-derived small RNA in plants have also been presented in other PSTVd studies [17,89,90].

A transcriptome analysis (by RNA-seq) of tomato “Heinz 1706” upon PSTVd^Int^ infection at 3 weeks post inoculation clearly showed activation of the plant immune response. This response includes, for example, elevated expression of genes encoding the mitogen-activated protein kinase 3 (MAPK3), PR1, 13 WRKY TFs, four calcium-dependent protein kinases (CDPKs), and 6 NBS-LRRs, which are involved in the recognition of pathogen avirulence determinants. The expression of genes encoding various receptor-like kinases (RLKs) were activated (50 genes) or repressed (15 genes) [23]. Our data also revealed increased transcript levels of genes related to various signaling pathways (Table S4). CDPKs are Ca^2+^-sensors that decode Ca^2+^ signals into specific physiological responses. Some of them are involved in phytohormone signaling pathways, thus influencing plant growth, development and stress responses [91]. Seven genes encoding CDPKs were up-regulated in S23 infection mostly at 17 and 49 dpi, but only three of them in the mild infection (including one that was down-regulated at 8–17 dpi). Additionally, we observed activation of genes encoding MAPK3 (M variant, 24 dpi) MAPK4 and MAPK7 (S23, 17 and 49 dpi), several WRKY TFs (Figure 5 and Table S4), and over 30 RLKs.

We also observed elevated expression of many genes for pathogenesis-related (PR) proteins, such as PR2, PR4, PR5, chitinases, or snakin-like proteins. Some of them were especially highly transcribed, e.g., FC was approximately 600 or 100 for the *PR5* gene in S23 infection at 17 dpi or in M at 24 dpi, respectively. The transcript levels of other defense/stress-related genes, such as those encoding thaumatin-like protein, thioredoxin, superoxide dismutase (SOD), and MLP-like proteins, were decreased. Interestingly, the gene for MLP-like protein 423-like was highly down-regulated at three time points, 8, 17, and 24 dpi, exclusively in PSTVd-M-infected plants. Two genes encoding glutathione S-transferase, which performs a number of functions including sequestration of toxins, mitigation of oxidative stress and possibly hormone response [92], were up-regulated in S23 (17–49 dpi) and one of them in M only at 24 dpi. An important part of plant response to biotic stress consists of ROS and ROS-scavenging enzymes. The expression of genes encoding peroxidases, which are enzymes implicated in the generation of ROS during pathogen defense was generally increased in S23-infected plants, while only three peroxidases were up-regulated in M infection at 24 dpi. The highly reactive nature of ROS can be harmful to host cell molecules, membranes and proteins. It has been proposed that the chlorotic symptoms of virus infection are the results, in full or part, of damage done to chlorophyll and/or chloroplasts by ROS [93]. The observed physiological changes in S23-infected tomato plants could have the same explanation. Moreover, genes for some enzymes that control ROS levels by detoxifying hydrogen peroxide and superoxide anions, such as SOD and thioredoxins, were down-regulated (Table S8).

Plant hormones regulate metabolic pathways related to plant growth, development and responses to biotic and abiotic stress. Published studies on gene expression during intermediate PSTVd infection revealed a complex array of changes affecting hormone signaling [43]. In our experiment, the expression levels of over 140 genes involved in hormone metabolism and signaling were altered (Table S5). Genes related to JA, ET and SA, which play major roles in regulating plant defense responses to various pathogens, were generally up-regulated, but overall, the expression of more genes was differentially regulated in S23 and, unlike in M infection, these genes retained altered expression at 49 dpi (Figure 6). ABA has a remarkable impact on plant defenses against various pathogens, including viruses [94]. Both ABA and SA influence callose deposition at the plasmodesmata (PD), a mechanism that limits cell-to-cell viral movement. The results obtained by Adkar-Purushothama and co-workers [17] indicate that targeting of tomato callose synthase (11-like and 12-like) mRNA by a small RNA—generated by RNA silencing from the virulence modulating region of different PSTVd variants—could contribute to inducing the typical symptoms associated with infection. Probes for the genes *CalS11-like* and *CalS12-like* were not presented on the microarray, but we observed down-regulation of *callose synthase 9-like* in plants with severe symptoms at 49 dpi. In another study of a different viroid-host combination (chrysanthemum stunt viroid (CSVd)-*Argyranthemum*), less callose deposition at PD was observed in the shoot apical meristem (SAM) of the Yellow Empire cultivar than in that of the Border Dark Red cultivar, and this effect was correlated with the ability of CSVd to spread to the uppermost cell layers of the apical dome and the youngest leaf primordia (1 and 2) in Yellow Empire. The observed differential ability of CSVd to invade the SAM was not correlated with disease symptom expression [95,96]. Plants control callose levels through the activity of hydrolytic enzymes called β-1,3-glucanases. ABA can enhance callose deposition in different tissues and organelles by transcriptional suppression of β-1,3-glucanases [94]. Induction of the genes encoding β-1,3-glucanases was observed during viral infections; this phenomenon resulted in the removal of callose and thereby facilitated viral trafficking [94]. In our microarray and nCounter analyses (Tables S1 and S2) the expression of β-1,3-glucanase genes was shown to be elevated continuously between 17–49 dpi and at 24 dpi in S23- and M-infected plants, respectively. Similarly, up-regulation of one β-1,3-glucanase gene was shown at 25 dpi with severe PSTVd strain [42]. In contrast to the two up-regulated genes connected with ABA biosynthesis during mild infection, nine genes in S23-infected plants were down-regulated, while genes related to ABA signaling were similarly (up- or down-) regulated in both infections. Some of these genes were also identified in a study of intermediate PSTVd strain infection in Rutgers plants and their expression was altered in a similar manner [43].

We observed down-regulation of genes involved in isoprenoid biosynthesis and variously altered regulation of genes related to brassinosteroids, auxins and cytokinins, depending on the particular genes and time after infection. It is clearly visible in our results that differential regulation of these genes occurs mainly at 17 and 49 dpi during S23 infection but at 24 dpi during M infection. The gibberellin-biosynthesis-related genes (*GA20ox1*, *GA20ox2*, *GA20ox3*, and *KAO*), however, did not fit this pattern. Elevated levels of these transcripts were observed at 24 dpi in S23-infected plants, a result that might seem to be at odds with the observed stunting. GA is known to regulate stem and hypocotyl elongation, fruit growth and development and seed development in tomato. However, the level of GA-related transcripts does not solely determine the hormone or protein level, especially because bioactive GA concentration is mainly controlled by a feedback mechanism regulating the expression of the biosynthetic genes *GA20ox* and *GA3ox* as well as by feedforward regulation of *GA2ox*, which codes for an inactivation enzyme [97]. Moreover, in our study, the expression of one of these genes, *GA20ox1*, was shown by microarray and nCounter analysis to be down-regulated at 17 dpi and up-regulated at 24 dpi. Hammond and Zhao [98] showed that overexpression of the protein kinase PKV (which could be induced by viroid infection) resulted in modification of tobacco development, including stunting. In these plants, the GA20ox1 and GA3β transcript levels were higher than those in taller non-transformed plants or those transformed with antisense *pkv*. Exogenous application of gibberellic acid reversed stunting in plants with overexpression of PKV, thus demonstrating feedback regulation; a low level of GA could result in the induction of these enzymes, whereas their gene expression would be repressed in plants containing higher levels of GA. Similarly, in an Arabidopsis study, the expression levels of *GA20ox1*, *GA20ox2*, *GA20ox3* and *GA3ox1* were highly elevated when bioactive GA levels were low but rapidly decreased when plants were treated with exogenous GA [99].

The stunting caused by viroid infection may be a result of the down-regulation of genes involved in the biosynthetic and signaling pathways of brassinosteroid hormones, which regulate cell division and elongation. Decreased levels of some transcripts coding for brassinosteroid synthesis enzymes during viroid infection were observed in other studies of tomato [43] and potato [45]. In our study, this down-regulation was only observed in plants with severe stunting, whereas only one gene involved in brassinosteroid synthesis was altered in plants infected with the M strain. Other hormones that influence plant growth include auxins (Table S5). Most genes related to auxin biosynthesis and signaling were down-regulated in S23-infected plants. In addition, S23 infection increased the expression of *GH3* genes, which inhibit plant growth by suppressing genes related to auxin biosynthesis and signaling and those encoding expansins [100,101]. On the other hand, regulation of *GH3* expression was not observed in M-infected plants. Expansins are involved in cell elongation by loosening cell wall components [102,103]. A previous study [104] demonstrated that the flat-top symptom observed in PSTVd^Int^U257A-infected tomato “Rutgers” was associated with inhibited cell growth correlated with the decreased expression of an expansin 2 gene (LeExp2). In our study, the expression level of this gene was decreased in S23-infected plants but the alteration was not statistically significant; however, a few genes encoding other expansins were decreased in S23 at 17 and/or 24 dpi. The expression level of *Exp4* at 17 dpi was confirmed by nCounter analysis. The observed increased levels of two other genes, *Exp1* and *Exp12*, at 49 dpi may be associated with the observed regrowth of the infected plants. In M-infected plants fewer expansin genes were altered. One gene encoding extensin, a cell-wall-localized hydroxyproline-rich glycoprotein that usually forms a cross-linked network with pectin to create a highly impassable barrier against pathogens, was differentially regulated in S23 and M infection (Table S7). A continuous decrease in the expression of pectinesterases in S23-infected plants, revealed previously by a microarray analysis of etrog citron (*Citrus medica* L.) infected with CEVd [46], could also have an impact on plant growth.

In summary, our analysis of gene expression allowed us to show differences between transcriptional changes in tomato leaves triggered by mild and severe PSTVd variants during infection development. Overall, the number of differentially expressed genes found in the severe infection was significantly higher than that in the mild infection, and over 74% of the DEGs were specific to S23 infection, in contrast to the M infection, which contained less than 20% unique DEGs. Finally, our transcriptome analysis has provided a list of differentially expressed genes in tomato during PSTVd infection, which could be useful for a more detailed study of tomato-viroid interaction.

## Supplementary Materials

The following are available online at http://www.mdpi.com/1999-4915/10/5/257/s1, Figure S1: Gene Ontology (GO) terms assigned to the complete list of the differentially expressed genes, Table S1: NanoString nCounter analysis of the selected DEGs, Table S2: Tomato DEGs affected by PSTVd-M and PSTVd-S23 infection, Table S3: GO term enrichments of different categories, Table S4: TF and PK genes in which expression was altered by PSTVd infection at particular time points, Table S5: Genes involved in plant hormone biosynthesis and signaling in which expression was altered by PSTVd infection, Table S6: Photosynthesis- and chloroplast-related genes in which expression was altered by PSTVd infection, Table S7: Differentially regulated cell-wall-related genes during PSTVd infection, Table S8. Differentially regulated defense-related genes during PSTVd infection.
