# Supplementary material for: Time-Course Microarray Analysis Reveals Differences between Transcriptional Changes in Tomato Leaves Triggered by Mild and Severe Variants of Potato Spindle Tuber Viroid
Source: Viruses. 2018 May 15;10(5):257. doi: 10.3390/v10050257 (PMC5977250; doi:10.3390/v10050257)
Supplement: Supplementary file 1 [file viruses-10-00257-s001.zip › Figure S1.pdf]

PSTVd-M

Biological Process

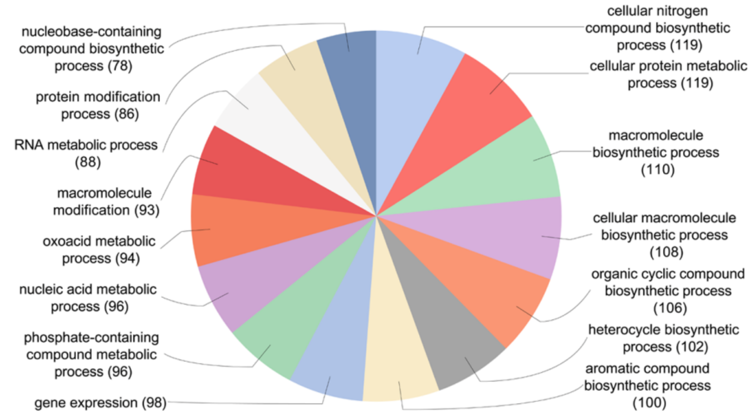

Cellular Component

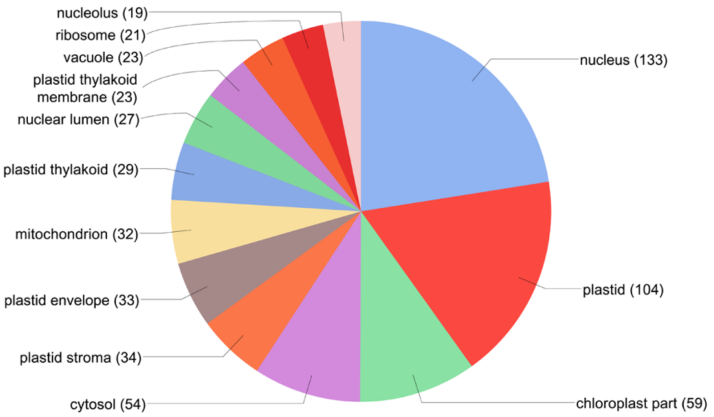

Molecular Function

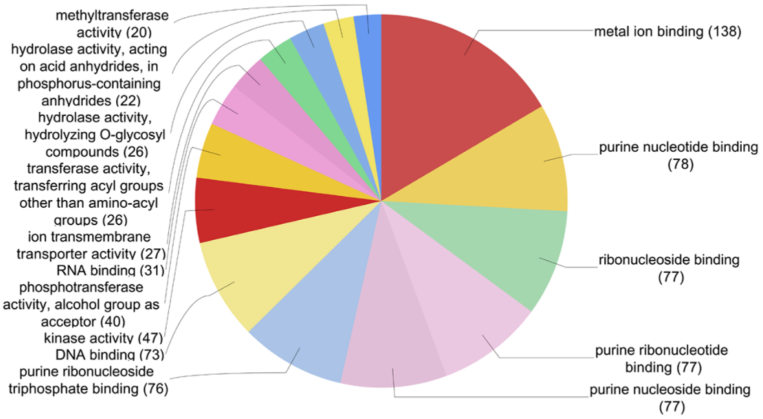

PSTVd-S23

Biological Process

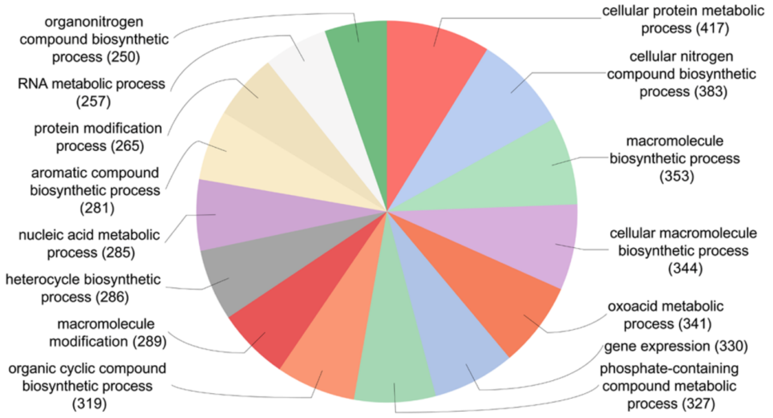

Cellular Component

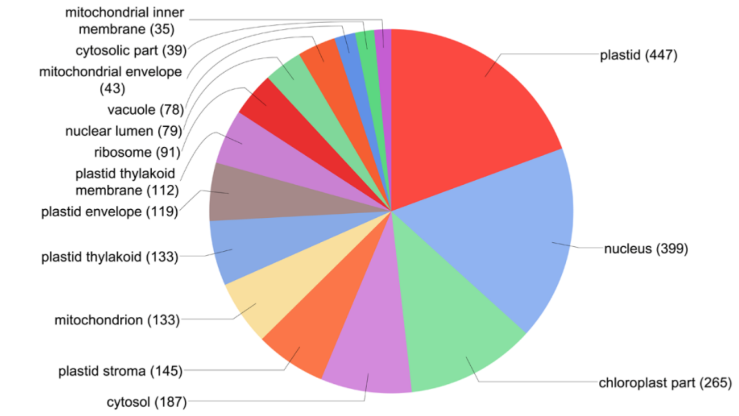

Molecular Function

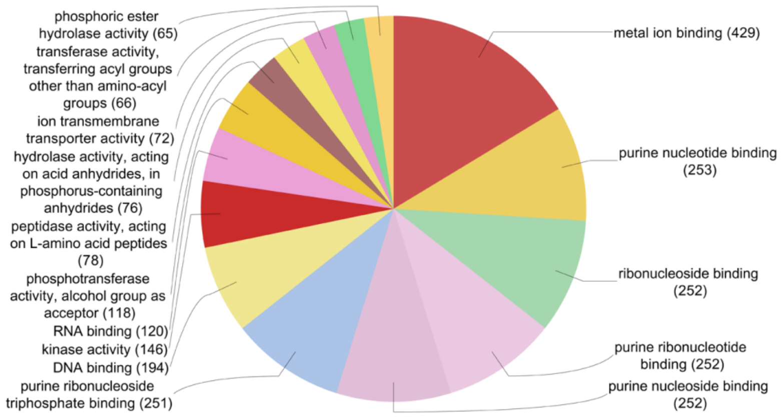

Figure S1. Gene Ontology (GO) terms assigned to the complete list of the differentially expressed genes. Pie charts represent the GO term distributions in plants infected with PSTVd-M (A) and PSTVd-S23 (B) as determined by Blast2GO under the biological process, molecular function and cellular component categories at GO level 5 with a 5-node score cutoff. The numbers in brackets represent the number of genes assigned to each GO term. 15 most abundant GO terms are presented.
